# Peripheral retinal cysts in presumed ocular toxocariasis

**DOI:** 10.1186/s12348-023-00357-y

**Published:** 2023-08-02

**Authors:** Rym Maamouri, Aida Jallouli, Olfa Béizig, Monia Cheour

**Affiliations:** Department of Ophthalmology, Habib Thameur Hospital, 3, Rue Ali Ben Ayed, 1089 Montfleury Tunis, Tunisia

**Keywords:** Peripheral retinal cysts, Ocular toxocariasis, Ultrasonic biomicroscopy

## Abstract

Typical clinical manifestations of ocular toxocariasis are central posterior granuloma, peripheral granuloma and chronic endophthalmitis. Herein we report the presence of peripheral subretinal cysts in two cases with a presumed ocular toxocariasis (OT).

## Photo essay

**Fig. 1:**
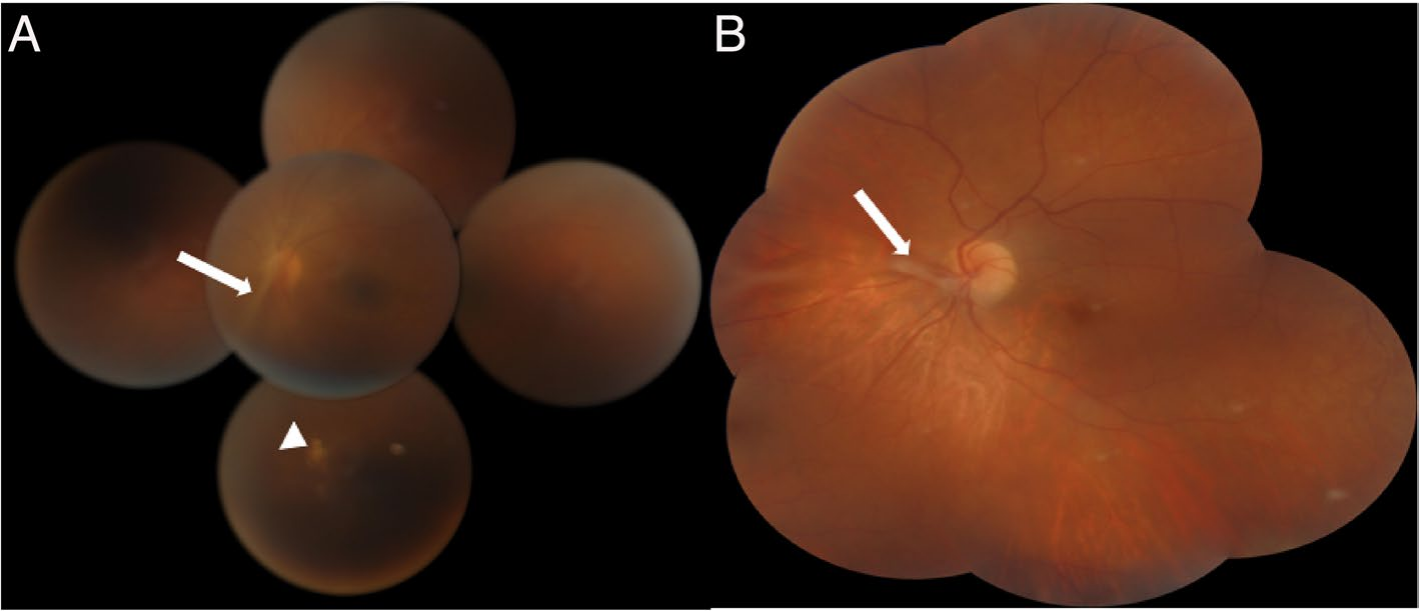
Montage color fundus photography in a 12-year-old patient (**A**) and in a 20-year-old patient (**B**) with ocular toxocariasis showing a vitreoretinal band running from the periphery toward the optic nerve head (white arrow). A peripheral chorioretinal scar is visible in the first patient (white arrowhead)

**Fig. 2:**
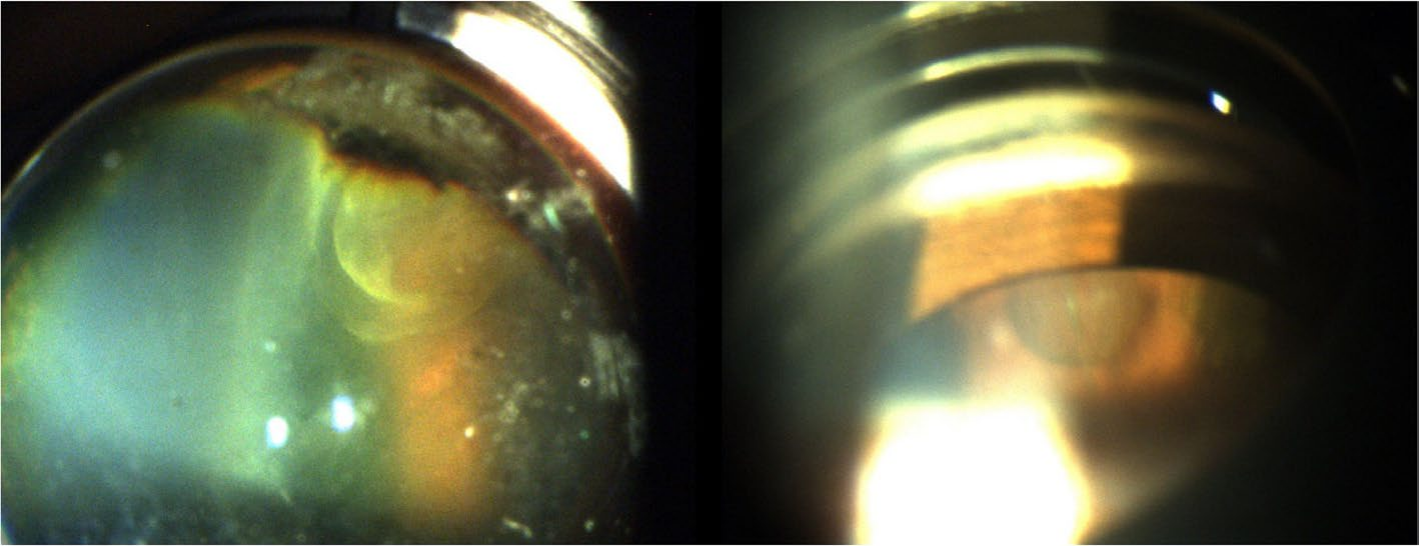
Slit-lamp photograph using a three-mirror lens showing peripheral rounded, well- circumscribed retinal cysts in both patients

**Fig. 3:**
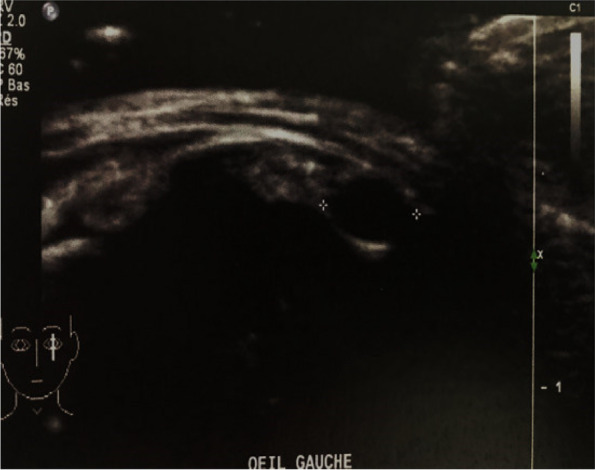
UBM showing in the second patient a peripheral cyst of 2.6 mm in diameter with thin wall localized between 4 o’clock and 5 ‘o’clock and a 2 mm peripheral cyst localized at 2 o’clock

Patients were aged 12 and 20 years old. They were referred to our department for the management of unilateral uveitis. This involved the left eye (OS) for both patients, and the duration of the uveitis before referral was one month for the first patient. The second patient was otherwise asymptomatic. Best corrected visual acuity of the affected left eyes was 20/200 and 20/50, respectively. Anterior segment examination showed the presence of granulomatous anterior uveitis with elevated intraocular pressure, posterior synechiae for the first patient and demonstrated normal results for the second patient. Fundus examination showed the presence of vitritis associated with a vitreoretinal band running from the periphery toward the optic nerve head. A peripheral chorioretinal scar is visible in the first patient. (Fig. 1). Careful examination demonstrated the presence of peripheral retinal cyst in OS for both patients (Fig. 2). Results of ocular examination of the right eye were unremarkable for both patients. Swept source optical coherence tomography showed the presence of macular edema for the first patient. Ultrasound biomicroscopy (UBM) confirmed the presence of peripheral cysts of 2.6 mm and 2 mm in size, respectively, in the second patient (Fig. 3).

Based on clinical presentation, the diagnosis of presumed OT was made and both patients underwent enzyme-linked immunosorbent assay (ELISA) serum test and complete blood count that came back negative with normal eosinophilia respectively for both patients.

Despite the fact that the clinical manifestations of OT are often characteristic, the diagnosis remains challenging, especially in a patient with normal eosinophilia and negative ELISA serology since it’s often repeatedly negative [1].

Peripheral vitreoretinal toxocariasis include vitreal membranes, toxocara granuloma, pseudocysts, thickening of the ciliary body, cystic formation and peripheral retinal detachment, and these lesions were mostly seen and described by UBM, which was considered as an additional diagnostic tool of OT [1, 2]. Pseudocystic formation were otherwise reported to be characteristic of OT [2, 3].

The origin of retinal cysts is not likely to be larva transformation since that during their migration they usually stop growing and end up dying in human tissue [4]. Therefore, some authors suggested that retinal cysts would be caused by a specific OT inflammatory process, often described with chronic uncontrolled intermediate uveitis, leading to vitreous shrinkage and traction resulting in pseudocystic aspect or peripheral retinoschisis [3, 4].

From these two cases, we emphasize the importance of a meticulous peripheral retinal examination in patients with OT in order to look for these peripheral characteristic cysts. In addition, it would be worthwhile to use ultrawide-field imaging and optical coherence tomography, when feasible, in order to ensure better view of the peripheral vitreoretinal changes in OT and provide a more detailed analysis enabling a more accurate diagnosis.

## Data Availability

The data used in that case report is available from the corresponding author on reasonable request.

## References

[1] Sharkey JA, McKay PS (1993). Ocular toxocariasis in a patient with repeatedly negative ELISA titre to Toxocara canis. Br J Ophthalmol.

[2] Tran VT, Lumbroso L, LeHoang P, Herbort CP (1999). Ultrasound biomicroscopy in peripheral retinovitreal toxocariasis. Am J Ophthalmol.

[3] Despommier D (2003). Toxocariasis: clinical aspects, epidemiology, medical ecology, and molecular aspects. Clin Microbiol Rev.

[4] Pichi F, Srivastava SK (2017). Nucci Paolo, Baynes K, Neri P, Lowder C Peripheral retinoschisis in intermediate uveitis. Retina.

